# The Application of Six Sigma to Assess the Analytical Performance of Plasma Proteins and Design a Risk‐Based Statistical Quality Control Strategy: A Multicenter Study

**DOI:** 10.1002/jcla.70080

**Published:** 2025-07-18

**Authors:** Ming Hu, Jiaping Wang, Huan Yang, Sugang Zang, Tingting Gao, Jian Zeng, Fumeng Yang

**Affiliations:** ^1^ Department of Laboratory Medicine People's Hospital of Donghai County Lianyungang People's Republic of China; ^2^ Department of Laboratory Medicine The Second Affiliated Hospital of Suzhou University Suzhou People's Republic of China; ^3^ Department of Laboratory Medicine Suqian City People's Hospital Suqian People's Republic of China; ^4^ Department of Laboratory Medicine Traditional Chinese Medicine Hospital of Fuxin Fuxin People's Republic of China; ^5^ Department of Laboratory Medicine Traditional Chinese Medicine Hospital of Lianyungang Lianyungang People's Republic of China; ^6^ Department of Laboratory Medicine Affiliated Lianyungang Clinical College of Nantong University Lianyungang People's Republic of China; ^7^ Department of Laboratory Medicine The Second People's Hospital of Lianyungang Affiliated with Kangda College of Nanjing Medical University Lianyungang People's Republic of China; ^8^ Department of Laboratory Medicine Lianyungang Clinical College, Jiangsu University and The Second People's Hospital of Lianyungang Lianyungang People's Republic of China; ^9^ Department of Laboratory Medicine Lianyungang Clinical College, Xuzhou Medical University and The Second People's Hospital of Lianyungang Lianyungang People's Republic of China

**Keywords:** plasma protein, quality control, quality goal index, six sigma

## Abstract

**Background:**

This study applied the six sigma model to evaluate plasma protein testing performance in six laboratories, with customized quality control programs and targeted improvements introduced where necessary.

**Methods:**

Internal quality control (IQC) and external quality assessment (EQA) data for plasma proteins were gathered from six laboratories. Sigma values for each analyte were determined based on the coefficient of variation (CV), bias, and total allowable error (TEa). Using six sigma performance verification charts, we calibrated analyte performance and, guided by Westgard sigma rules, batch length, and quality goal index (QGI), developed laboratory‐specific quality control schemes and improvement plans.

**Results:**

Despite standardized platforms and reagents, sigma values showed significant inter‐laboratory variation, with some differences also observed within labs at varying analyte concentrations. For projects with sigma < 6, tailored quality control measures were implemented, leading to marked performance improvements.

**Conclusion:**

The six sigma model provides an objective framework for evaluating plasma protein test performance and enhancing quality. It enables quantitative assessment of laboratory management and supports the development and implementation of customized, risk‐based statistical quality control (SQC) strategies and improvement measures across multiple laboratory systems.

## Graphical abstract

The six sigma model can objectively assess the analytical performance of plasma proteins. Different control concentrations yield different sigma values, which are visually displayed through standardized sigma performance verification charts. The six sigma model enables the development of personalized quality control strategies for each assay. Based on the Six Sigma model and the Quality Goal Index (QGI), precise improvement measures can be provided for assays with suboptimal analytical performance (sigma < 6).

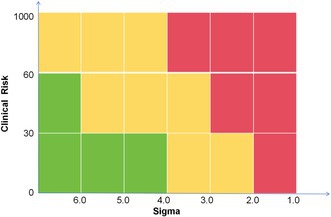

**Abbreviations**
ASO: antistreptolysin O
C3: complement 3
C4: complement 4
CRP: C‐reactive protein
CV: coefficient of variation
EFLM: European Federation of Clinical Chemistry and Laboratory Medicine
EQA: external quality assessment
IgA: immunoglobulin A
IgG: immunoglobulin G
IgM: immunoglobulin M
IQC: internal quality control
QGI: quality goal index
RF: rheumatoid factors
TEa: total allowable error

## 1 Introduction

The Westgard multi‐rule system, a classic quality control method widely used in clinical laboratories [1], aids in identifying testing errors, distinguishing systematic from random errors, and guiding corrective measures accordingly. However, its limitation lies in the lack of customization for individual tests, as it applies a uniform set of rules across all analytes. The six sigma management model addresses these limitations by enabling tailored quality control strategies.

As laboratory quality management has evolved, the six sigma model has largely supplanted traditional Westgard rules, becoming a key approach in clinical labs worldwide [2, 3]. Developed by Motorola engineer Bill Smith [4], six sigma is a strategic method for quality improvement, targeting error reduction and process consistency [5]. Since Nevalainen et al. [6] first applied it to laboratory medicine in the early 21st century, six sigma has effectively transformed lab error rates into sigma metrics for performance evaluation. This model has proven successful across industries, including Motorola, Dell, and Siemens, and is now widely recognized in laboratory medicine for establishing risk‐based, individualized statistical quality control (SQC) strategies [7]. The six sigma model, combined with inter‐laboratory quality assessment and internal quality control, offers robust guidance for quality enhancement and helps establish targeted quality control protocols, thus becoming a focal point in laboratory quality management research [8, 9, 10].

Clinical laboratory quality control (QC) plays a critical role in ensuring the accuracy and reliability of test results. Sigma metrics, a key metric for evaluating laboratory analytical performance, are widely used in various quality control activities. It assesses the performance of laboratory testing methods by taking into account both measurement imprecision and bias. As laboratory quality management has evolved, the methods for calculating sigma values have undergone several improvements, transitioning from traditional Shewhart control charts to more advanced trend detection methods, such as Exponentially Weighted Moving Average (EWMA) and Cumulative Sum (CUSUM) charts.

In recent years, with the introduction of risk management concepts, the application of sigma value has expanded significantly. Notably, incorporating parameters such as Maximum Allowable Measurement Uncertainty (MAU) into sigma value calculations has led to more precise quality control standards [11]. Furthermore, sigma scores have gained increasing importance in patient safety and error management. Research has shown that the severity of laboratory errors is closely linked to sigma value, particularly when critical parameters such as blood gas analysis, cardiac markers, and coagulation function tests are involved. Errors in sigma value can lead to significant patient risks [12].

Six sigma has been extensively applied to evaluate analyte performance in clinical biochemistry, hematology, immunology, and more, supporting individualized quality control in laboratories [13, 14, 15, 16]. In this study, we aim to assess plasma protein testing performance in six laboratories using the six sigma model and to develop individualized quality control strategies for each laboratory.

## 2 Materials and Methods

### 2.1 Materials

#### 2.1.1 Sample Source and Transportation

The internal and external quality control samples used in this study were lyophilized control materials provided by the National Center for Clinical Laboratories (NCCL) of China. All samples were transported under strict cold‐chain conditions at 2°C–8°C to ensure their biological stability. Upon arrival, samples were reconstituted and stored according to standardized procedures across all six laboratories to minimize pre‐analytical variability. The lyophilized quality control materials were reconstituted in each laboratory according to the manufacturer's guidelines. Each laboratory followed the same preparation procedures to ensure consistency, including careful reconstitution to avoid bubbles and ensure complete dissolution.

### 2.2 Instruments and Reagents

This study analyzed quality control data from six laboratories (designated A through F) using IMMAGE 800 immunochemistry systems (Beckman Coulter, USA). Original Beckman Coulter reagents and calibrators were used, along with American Bio‐Rad quality control products in low (Lot: 68981) and high (Lot: 68983) concentration batches. Additionally, the external quality assessment (EQA) samples, comparable to the internal quality control (IQC) levels, were obtained from the National Center for Clinical Laboratories of China.

All participating laboratories used the same brand of IQC materials, and their concentrations were comparable to the target concentrations of the EQA samples. To ensure the valid integration of bias and coefficient of variation (CV) values, we only used samples with comparable concentrations for bias calculations. The concentrations of the samples were carefully reviewed to ensure that the EQA sample concentrations matched those of the IQC samples within a reasonable range, thus ensuring the validity of combining bias and CV values.

### 2.3 Analytes

The plasma proteins analyzed included immunoglobulin G (IgG), immunoglobulin A (IgA), immunoglobulin M (IgM), complement 3 (C3), complement 4 (C4), C‐reactive protein (CRP), anti‐streptolysin O (ASO), and rheumatoid factor (RF).

### 2.4 Analytical Methods

#### 2.4.1 Total Allowable Error (TEa)

The TEa was determined according to standards from the National Health Commission's Clinical Laboratory Center inter‐laboratory quality assessments.

Bias and sigma calculations were performed at specific QC levels to provide a comprehensive assessment of laboratory performance. Bias refers to the difference between the observed values and the target values, and calculating the bias at different QC levels helps identify systematic errors at each quality control level. On the other hand, sigma quantifies overall analytical performance by evaluating the standard deviation distance between the process and the target, thus assessing the laboratory's overall process capability. By considering both bias and sigma, it is possible to identify specific sources of error, evaluate the overall process capability, and develop more effective and personalized quality control strategies.

To ensure the validity of bias calculations, samples must be interchangeable. Therefore, we confirmed with the EQA provider that the EQA samples for the eight analytes used in this study were compatible with the IMMAGE 800 analyzer. Based on this confirmation, bias calculations were performed.

The EQA samples used in this study were selected to match the analyte concentrations of the IQC samples used in each laboratory. This approach ensures that both IQC and EQA samples are tested under similar analytical conditions. By aligning the concentrations, we aimed to minimize the impact of concentration differences on performance metrics, ensuring more accurate comparisons between internal and external quality control data. This method enabled a more reliable assessment of each laboratory's performance and helped identify any discrepancies in the results.

#### 2.4.2 Imprecision (CV)

CV values for IQC measurements of special proteins were gathered from February to December 2023 across all six laboratories, each following the same IQC scheme. Target values for each analyte were established based on the average of multiple valid measurements, using manufacturer‐provided values as a reference. CVs for two IQC levels of special protein analytes were calculated from each laboratory's actual measurement results.

#### 2.4.3 Bias

Bias was calculated based on target values derived from the median results of 373 independent laboratories participating in the National Inter‐laboratory Quality Assessment (EQA) program. All laboratories used the same analytical system and reagent kit, ensuring consistency in testing methodology. Although the target values were not obtained through reference methods, the median value is widely accepted as a reliable proxy for the true value in large‐scale EQA settings. To minimize the impact of short‐term variability, long‐term average bias from multiple EQA cycles was used.

The average bias for each analyte at different concentration levels was calculated using inter‐laboratory quality assessment data from the National Health Commission's Clinical Laboratory Center. EQA samples with analyte concentrations comparable to those in the IQC materials were selected, and the average bias for each target level was determined based on the bias of individual data points [17, 18].

$${\text{Bias}}_{\text{average}}=\frac{\left|{\text{Bias}}_{1}\right|+\left|{\text{Bias}}_{2}\right|+\left|{\text{Bias}}_{3}\right|+\cdots +\left|{\text{Bias}}_{n}\right|}{n}$$

Each EQA sample was measured five times in repetition, and bias was calculated as the difference between the average of these five measurements and the reference value. Repeated measurements help reduce analytical variability, ensuring that the bias calculation is both more accurate and reliable.

In this study, two QGI values were used for each parameter. The first QGI value was used to assess the analytical performance of the parameter, such as imprecision and trueness, while the second QGI value was used to evaluate its relevance in clinical applications. This approach allows for a comprehensive evaluation of each parameter's quality from different perspectives, ensuring its reliability in both laboratory analysis and clinical practice.

However, when selecting the outcome, we chose a single outcome indicator that most accurately reflects our research objective and holds significant clinical value. This strategy ensures the simplicity and clarity of the outcome results, while maintaining rigorous quality control in the study.

#### 2.4.4 Quality Goal Index (QGI)

QGI was used as a supplementary tool to identify the primary source of performance deviations when sigma values were below 3. The QGI was calculated using the formula QGI = Bias/(0.5 × CV). A QGI < 0.8 indicates imprecision‐dominant error, a QGI > 1.2 indicates trueness, and a QGI between 0.8 and 1.2 suggests that both factors contribute [19].

Figure 2 illustrates the risk‐based quality control model. This model takes into account both analytical performance (measured by Sigma values) and clinical risk (the potential severity of harm associated with the tested parameter). Specifically, a high Sigma value indicates a reliable measurement system with infrequent quality control, while a low Sigma value indicates an unreliable measurement system, necessitating more frequent quality control. Additionally, clinical risk factors are incorporated to adjust the frequency of quality control based on the potential harm that measurement errors may cause to patients.

**FIGURE 1:**
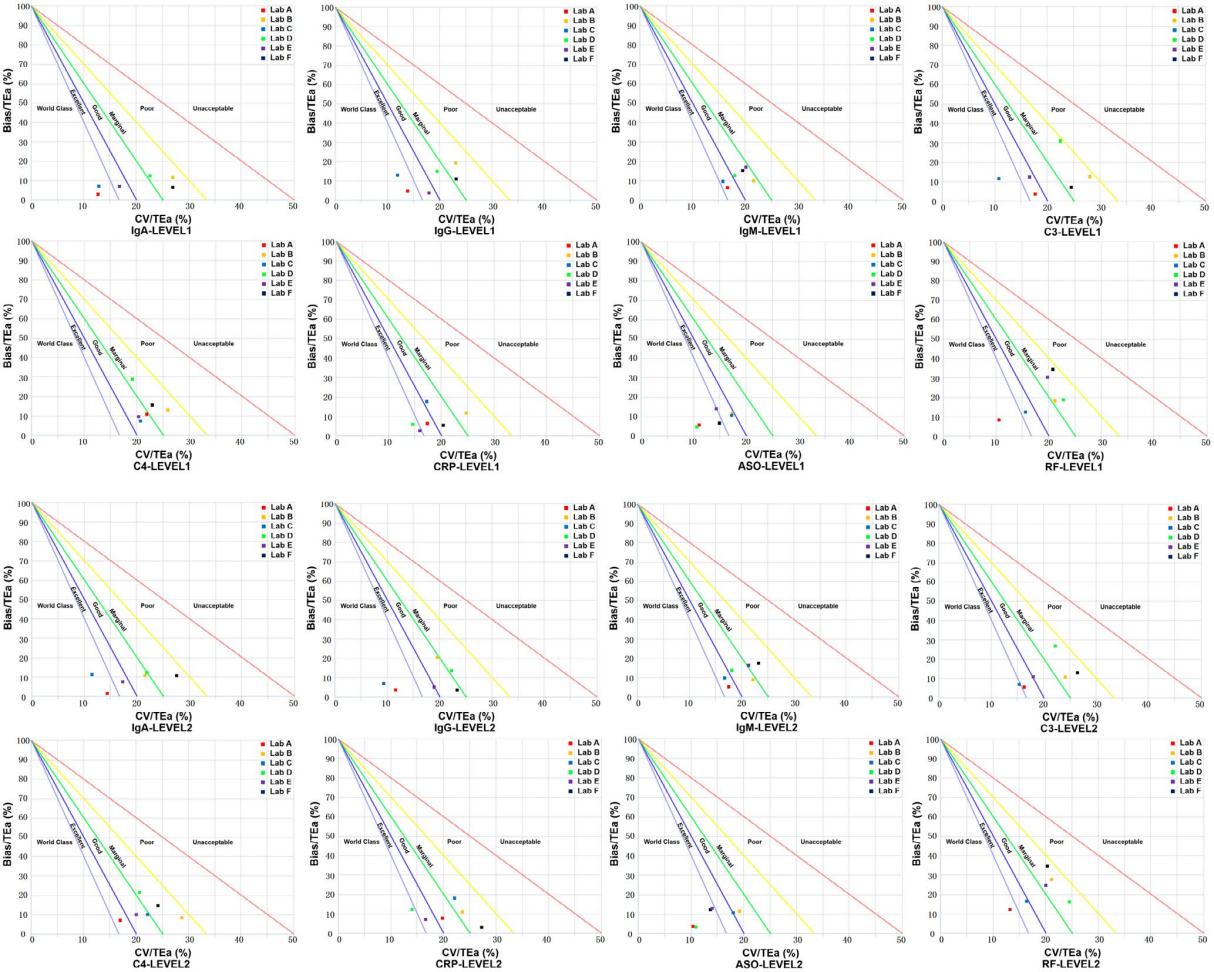
Standardized sigma performance verification of plasma proteins for QC 1 and QC 2. The five lines in the chart represent negative sigma values, indicating that when the testing method aligns with any of these lines, the negative slope corresponds to the sigma value of the analytical performance. The *x*‐axis represents the coefficient of variation (CV) relative to total allowable error (TEa) (%), indicating imprecision, while the *y*‐axis represents bias relative to total allowable error (TEa) (%), indicating trueness.

The risk‐based quality control selection model (Figure 1) combines analytical performance (sigma values) and clinical risk (potential severity of harm). This model is now a two‐dimensional framework, where the performance of the measurement system (Sigma) and the clinical impact (severity of harm) together determine the frequency of quality control. This model reflects the latest quality control approach proposed by Çubukçu et al. [11], offering a more comprehensive decision‐making framework for clinical laboratory quality management.

#### 2.4.5 Sigma Metric Calculation

Using software from the National Health Commission's Clinical Laboratory Center, the TEa and CV values for each special protein assay were entered to generate standardized sigma performance charts [20]. The sigma metric was calculated as sigma = (TEa—Bias)/CV. Performance was then categorized by sigma value: sigma < 2 (unacceptable), 2 ≤ sigma < 3 (poor), 3 ≤ sigma < 4 (marginal), 4 ≤ sigma < 5 (good), 5 ≤ sigma < 6 (excellent), and sigma ≥ 6 (world‐class).

#### 2.4.6 Designing Risk‐Based SQC Strategies and Improvement Measures

Using the Westgard sigma rules flowchart with batch length (Figure 1), the running size and analytical performance of plasma proteins guide laboratories in developing risk‐based SQC strategies [21].

## 3 Results

### 3.1 Sigma Metrics for Plasma Protein Assays in Six Laboratories

This study assessed the sigma metrics of plasma protein analytes across six laboratories using the six sigma model. Sigma values varied by analyte concentration within laboratories. For example, CRP in Laboratory A had sigma values of 5.38 at IQC Level 1 and 4.67 at Level 2. Despite using the same detection platform, performance differences were evident across labs; IgA achieved six sigma in Labs A and C, a five sigma in Lab E, and only 3–4 sigma in Labs B, D, and F. Overall, sigma distributions were as follows: 25.0% at sigma ≥ 6, 16.7% within 5 ≤ sigma < 6, 22.9% within 4 ≤ sigma < 5, and 35.4% within 3 ≤ sigma < 4. Sigma metrics of plasma protein analytes are detailed in Table 1.

**TABLE 1:** Sigma metrics of plasma proteins assays at two quality control levels in six laboratories.

Analyte	QC level	TEa (%)	Lab A		Lab B		Lab C		Lab D		Lab E		Lab F	
			CV	Sigma	CV	Sigma	CV	Sigma	CV	Sigma	CV	Sigma	CV	Sigma
IgA	Level 1	25	3.19	7.65	6.72	3.28	3.22	7.20	5.65	3.85	4.19	5.58	6.78	3.44
	Level 2	25	3.59	6.84	5.39	4.14	2.98	7.43	5.47	3.99	4.36	5.30	6.92	3.22
IgG	Level 1	25	3.46	6.87	5.83	3.46	3.03	7.25	4.87	4.36	4.46	5.33	5.85	3.79
	Level 2	25	2.94	8.20	5.38	3.60	2.34	9.95	5.59	3.89	4.76	4.98	5.73	4.19
IgM	Level 1	25	4.18	5.62	5.39	4.18	3.97	5.71	4.53	4.84	5.02	4.15	4.88	4.37
	Level 2	25	4.33	5.51	5.52	4.10	4.10	5.50	4.52	4.78	5.31	3.91	5.73	3.62
C3	Level 1	25	4.34	5.56	6.96	3.10	2.76	7.97	5.64	3.07	4.13	5.29	6.17	3.76
	Level 2	25	4.14	5.70	6.04	3.68	3.83	6.04	5.57	3.29	4.50	4.94	6.71	3.25
C4	Level 1	25	5.51	4.03	6.46	3.37	5.25	4.38	4.77	3.73	5.12	4.40	5.71	3.69
	Level 2	25	4.35	5.35	7.25	3.17	5.53	4.07	5.18	3.79	5.03	4.47	5.99	3.57
CRP	Level 1	25	4.35	5.38	6.18	3.56	4.38	4.70	3.66	6.41	4.01	6.07	5.11	4.62
	Level 2	25	4.96	4.67	5.87	3.79	5.53	3.72	3.50	6.26	3.87	6.08	6.81	3.55
ASO	Level 1	25	2.78	8.57	4.35	5.11	4.38	5.09	2.71	8.84	3.63	6.03	3.72	6.27
	Level 2	25	2.61	9.30	4.87	4.55	4.51	4.98	2.71	8.96	3.49	6.27	3.43	6.41
RF	Level 1	25	2.70	8.52	5.33	3.84	3.95	5.52	5.68	3.60	4.95	3.51	5.24	3.13
	Level 2	25	3.30	6.60	5.15	3.51	4.08	5.11	5.91	3.53	5.03	3.74	5.21	3.13

*Note:* TEa: allowable total error, which was derived from the EQA standard of China.

Abbreviations: ASO, anti‐streptolysin O; C3, complement 3; C4, complement 4; CRP, C‐reactive protein; IgA, immunoglobulin A; IgG, immunoglobulin G; IgM, immunoglobulin M; RF, rheumatoid factor.

### 3.2 Standardized Sigma Performance Verification Charts

Standardized sigma verification charts (Figure 1) provide a visual summary of analyte performance across control levels in each lab, highlighting method trueness and identifying improvement needs to uphold quality control. For example, the IgA analysis project at Laboratory A is at a “world‐class” level, as shown in Figure 1.

### 3.3 Risk‐Based SQC Strategy Development

**FIGURE 2:**
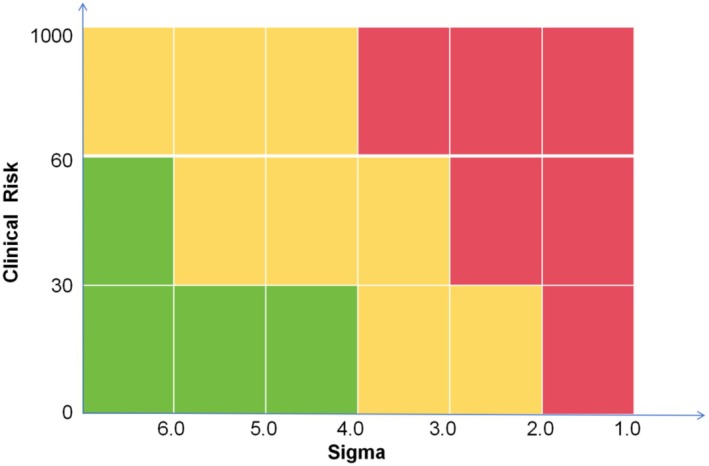
Risk matrix heatmap based on sigma values and clinical risk levels. The different colored blocks (as indicated by the gradient in the legend) provide a visual representation of the risk level in each zone. The icons on the chart denote the quality‐control recommendations for each zone: Green: Routine QC recommended; Yellow: Increase QC frequency; Red: Method replacement or immediate intervention required.

The QGI was calculated for each laboratory based on the laboratory data. Individualized quality control strategies were then developed for each laboratory and analyte, using risk matrix heatmap based on sigma values and clinical risk levels (Figure 2). To ensure the effectiveness of quality improvement, we applied more stringent quality control rules based on the sigma values generated at different concentration levels of the same assay. For example, in the IgA analysis project at Laboratory B, the sigma values at two concentration levels are 3.28 and 4.14. To ensure the effectiveness of the quality control measures, the lower sigma value is chosen as the basis for the quality control plan. Consequently, the control chart rules 13s/22s/R4s/41S/8X are selected, with parameters set to *N* = 4, *R* = 2, and *S* = 45. These strategies were tailored to the specific needs of each laboratory, with the corresponding quality management practices outlined (Table 2).

**TABLE 2:** Risk‐based SQC strategies of plasma proteins assays in six laboratories.

Analyte	Risk‐based SQC strategies					
	Lab A	Lab B	Lab C	Lab D	Lab E	Lab F
IgA	1_3s_ with *N* = 2 and *S* = 1000	1_3s_/2_2s_/R_4s_ /4_1s_/8_x_ with *N* = 4/R = 2 and *S* = 45	1_3s_ with *N* = 2 and *S* = 1000	1_3s_/2_2s_/R_4s_ /4_1S_/8_x_ with *N* = 4/R = 2 and *S* = 45	1_3s_/2_2s_/R_4s_ with *N* = 2 and *S* = 450	1_3s_/2_2s_/R_4s_ /4_1s_/8_x_ with *N* = 4/R = 2 and *S* = 45
IgG	1_3s_ with *N* = 2 and *S* = 1000	1_3s_/2_2s_/R_4s_ /4_1s_/8_x_ with *N* = 4/R = 2 and *S* = 45	1_3s_ with *N* = 2 and *S* = 1000	1_3s_/2_2s_/R_4s_ /4_1s_/8_x_ with *N* = 4/R = 2 and *S* = 45	1_3s_/2_2s_/R_4s_ with *N* = 2 and *S* = 450	1_3s_/2_2s_/R_4s_ /4_1s_/8_x_ with *N* = 4/R = 2 and *S* = 45
IgM	1_3s_/2_2s_/R_4s_ with *N* = 2 and *S* = 450	1_3s_/2_2s_/R_4s_ /4_1s_ with *N* = 4 and *S* = 200	1_3s_/2_2s_/R_4s_ with *N* = 2 and *S* = 450	1_3s_/2_2s_/R_4s_ /4_1s_ with *N* = 4 and *S* = 200	1_3s_/2_2s_/R_4s_ /4_1s_/8_x_ with *N* = 4/R = 2 and *S* = 45	1_3s_/2_2s_/R_4s_ /4_1s_/8_x_ with *N* = 4/R = 2 and *S* = 45
C3	1_3s_/2_2s_/R_4s_ with *N* = 2 and *S* = 450	1_3s_/2_2s_/R_4s_ /4_1s_/8_x_ with *N* = 4/R = 2 and *S* = 45	13s with *N* = 2 and *S* = 1000	1_3s_/2_2s_/R_4s_ /4_1s_/8_x_ with *N* = 4/R = 2 and *S* = 45	1_3s_/2_2s_/R_4s_ /4_1s_ with *N* = 4 and *S* = 200	1_3s_/2_2s_/R_4s_ /4_1s_/8_x_ with *N* = 4/R = 2 and *S* = 45
C4	1_3s_/2_2s_/R_4s_ /4_1s_ with *N* = 4 and *S* = 200	1_3s_/2_2s_/R_4s_ /4_1s_/8_x_ with *N* = 4/R = 2 and *S* = 45	1_3s_/2_2s_/R_4s_ /4_1s_ with *N* = 4 and *S* = 200	1_3s_/2_2s_/R_4s_ /4_1s_/8_x_ with *N* = 4/R = 2 and *S* = 45	1_3s_/2_2s_/R_4s_ /4_1s_ with *N* = 4 and *S* = 200	1_3s_/2_2s_/R_4s_ /4_1s_/8_x_ with *N* = 4/R = 2 and *S* = 45
CRP	1_3s_/2_2s_/R_4s_ /4_1s_ with *N* = 4 and *S* = 200	1_3s_/2_2s_/R_4s_ /4_1s_/8_x_ with *N* = 4/R = 2 and *S* = 45	1_3s_/2_2s_/R_4s_ /4_1s_/8_x_ with *N* = 4/R = 2 and *S* = 45	1_3s_ with *N* = 2 and *S* = 1000	1_3s_ with *N* = 2 and *S* = 1000	1_3s_/2_2s_/R_4s_ /4_1s_/8_x_ with *N* = 4/R = 2 and *S* = 45
ASO	1_3s_ with *N* = 2 and *S* = 1000	1_3s_/2_2s_/R_4s_ /4_1s_ with *N* = 4 and *S* = 200	1_3s_/2_2s_/R_4s_ /4_1s_ with *N* = 4 and *S* = 200	1_3s_ with *N* = 2 and *S* = 1000	1_3s_ with *N* = 2 and *S* = 1000	1_3s_ with *N* = 2 and *S* = 1000
RF	1_3s_ with *N* = 2 and *S* = 1000	1_3s_/2_2s_/R_4s_ /4_1s_/8_x_ with *N* = 4/R = 2 and *S* = 45	1_3s_/2_2s_/R_4s_ with *N* = 2 and *S* = 450	1_3s_/2_2s_/R_4s_ /4_1s_/8_x_ with *N* = 4/R = 2 and *S* = 45	1_3s_/2_2s_/R_4s_ /4_1s_/8_x_ with *N* = 4/R = 2 and *S* = 45	1_3s_/2_2s_/R_4s_ /4_1s_/8_x_ with *N* = 4/R = 2 and *S* = 45

*Note: N* = levels of quality control, *S* = number of patient samples run between quality control samples, *R* = frequency of the indoor quality control. These IQC programs were the new IQC programs instituted after reviewing the sigma data.

To further investigate the factors influencing analytical performance, we assessed the Quality Goal Index (QGI) of plasma protein assays with sigma metrics below 6 to identify whether imprecision, trueness, or both were contributing to performance deviations. For example, in Lab B's IgG assay, trueness was the primary limiting factor, necessitating improvements in calibration protocols and participation in external quality assessment programs. Similarly, Lab D's C3 and Lab F's RF assays exhibited significant bias, highlighting the need for enhanced calibration, imprecision, and reference material traceability. Conversely, Lab B and Lab F's IgA assays showed QGI values indicative of imprecision issues, requiring strengthened internal quality control, optimized reagent handling, and refined analytical workflows to reduce variability. Additionally, some assays, such as IgA in Lab D and C4 in Lab F, exhibited a combination of imprecision and trueness concerns, warranting a comprehensive approach to improving both imprecision and accuracy. These findings emphasize the necessity of laboratory‐specific quality enhancement strategies based on QGI analysis, ensuring improved reliability and consistency in plasma protein assays across different laboratories. The detailed improvement plans for each analyte are outlined in Table 3.

**TABLE 3:** The quality goal index and quality improvement measures for plasma proteins assays with sigma metrics < 6.

Analyte	Laboratory	Sigma metrics		QGI		Improvement measures
		Level 1	Level 2	Level 1	Level 2	
IgA	Lab B	3.28	4.14	0.66	0.75	Imprecision
IgA	Lab D	3.85	3.99	0.86	0.87	Imprecision and trueness
IgA	Lab E	5.58	5.30	0.57	0.65	Imprecision
IgA	Lab F	3.44	3.22	0.38	0.59	Imprecision
IgG	Lab B	3.49	4.09	1.24	1.59	Trueness
IgG	Lab D	4.36	3.89	1.15	0.87	Imprecision and trueness
IgG	Lab E	5.33	4.98	0.41	0.40	Imprecision
IgG	Lab F	3.79	4.19	0.73	0.25	Imprecision
IgM	Lab A	5.62	5.51	0.54	0.4	Imprecision
IgM	Lab B	4.18	4.10	0.68	0.64	Imprecision
IgM	Lab C	5.71	5.50	0.89	0.90	Imprecision and trueness
IgM	Lab D	4.84	4.78	1.03	1.13	Imprecision and trueness
IgM	Lab E	4.15	3.91	1.25	1.20	Trueness
IgM	Lab F	4.37	3.62	1.12	1.12	Imprecision and trueness
C3	Lab A	5.56	5.70	0.30	0.51	Imprecision
C3	Lab B	3.10	3.68	0.73	0.69	Imprecision
C3	Lab D	3.07	3.29	2.05	1.80	Trueness
C3	Lab E	5.29	4.94	1.15	0.92	Imprecision and trueness
C3	Lab F	3.76	3.25	0.43	0.72	Imprecision
C4	Lab A	4.03	5.35	0.76	0.59	Imprecision
C4	Lab B	3.37	3.17	0.75	0.42	Imprecision
C4	Lab C	4.38	4.07	0.58	0.68	Imprecision
C4	Lab D	3.73	3.79	2.28	1.55	Trueness
C4	Lab E	4.40	4.47	0.72	0.75	Imprecision
C4	Lab F	3.69	3.57	1.04	0.94	Imprecision and trueness
CRP	Lab A	5.38	4.67	0.55	0.56	Imprecision
CRP	Lab B	3.56	3.79	0.73	0.70	Imprecision
CRP	Lab C	4.70	3.72	1.51	1.20	Trueness
CRP	Lab F	4.62	3.55	0.41	0.18	Imprecision
ASO	Lab B	5.11	4.55	0.95	0.87	Imprecision and trueness
ASO	Lab C	5.09	4.98	0.92	0.84	Imprecision and trueness
RF	Lab B	3.84	3.51	1.28	2.02	Trueness
RF	Lab C	5.52	5.11	1.22	1.52	Trueness
RF	Lab D	3.60	3.53	1.20	1.04	Imprecision and trueness
RF	Lab E	3.51	3.74	2.31	1.85	Trueness
RF	Lab F	3.13	3.13	2.46	2.51	Trueness

Abbreviations: ASO, anti‐streptolysin O; C3, complement 3; C4, complement 4; CRP, C‐reactive protein; IgA, immunoglobulin A; IgG, immunoglobulin G; IgM, immunoglobulin M; QGI, quality goal index; RF, rheumatoid factor.

Although some CLSI documents suggest ignoring bias in the absence of reference methods, all 373 laboratories in this study used a unified platform and reagent. In Chinese EQA practice, median target values have been widely applied. Retaining bias helps assess systematic errors and enhances the clinical applicability of the Sigma evaluation.

While the QGI is not universally recognized in international standards, it is frequently used in national EQA initiatives in China. Therefore, the QGI metric was employed in this study as a supporting indicator rather than as a standalone decision‐making tool.

## 4 Discussion

The analysis of sigma values in this study revealed that low sigma scores in several laboratories were primarily due to imprecision or bias. For example, in Lab A, the sigma score for IgA was below 3, primarily due to high CV values, indicating significant imprecision. To address this, we recommend improving calibration procedures and optimizing reagent handling protocols to enhance imprecision. Conversely, in Lab D, low sigma scores for C3 and RF were primarily due to high bias, suggesting that improvements in calibration accuracy and better traceability of reference materials are necessary. Therefore, tailored quality control measures should be developed to target these specific deficiencies. Although all laboratories used the same immunochemistry platform (IMMAGE 800) and reagents from the same manufacturer, sigma values varied significantly across sites. This finding highlights the importance of localized factors, such as operator expertise, calibration, and equipment maintenance, in determining laboratory performance. The variability in sigma values is consistent with findings from other studies [22, 23], which show that, despite the use of standardized reagents and equipment, multi‐center analyses still reveal performance differences. These results emphasize that, even in a controlled environment, external factors such as training and procedural consistency can influence outcomes. The practical implication of this finding is that laboratories should not only rely on external validation data but also focus on internal factors, such as staff training and quality assurance practices, to reduce variability and improve consistency.

Although all laboratories followed standardized protocols, subtle differences in sample handling, storage, or preparation could have introduced variability, particularly in terms of imprecision. Variations in the reconstitution process, temperature fluctuations during storage, or inconsistencies in sample aliquoting could affect the final measurement results, thus impacting the overall sigma values. These factors may have significantly contributed to the inter‐laboratory variability observed in this study. Additionally, differences in calibration practices across laboratories could have further influenced the sigma scores.

Although we used two QGI values to assess the quality of each parameter—one for analytical performance evaluation and the other for clinical relevance—we ultimately selected a single outcome indicator as the primary objective of the study. This outcome was chosen due to its high clinical relevance in predicting patient outcomes and its clear reflection of the clinical goals of our research. We believe that this approach effectively balances laboratory quality control with clinical applicability, providing clinicians with easily interpretable results while maintaining a multidimensional evaluation of parameter quality.

In this study, two sigma values were calculated for each test: one based on imprecision (CV) and the other based on bias. The use of two sigma values reflects the fact that overall test performance is influenced by both random error (imprecision) and systematic error (bias). The sigma based on imprecision reflects the random variation in test results, while the sigma based on bias quantifies the systematic deviation between test results and the true value.

To ensure the most conservative assessment of laboratory performance, we selected the lower sigma value, ensuring that even in cases where one source of error (imprecision or bias) is more prominent, the laboratory still meets stringent performance requirements. This approach aligns with standard practices in the literature, where the lower sigma value is typically chosen to ensure optimal laboratory performance.

In our study, the risk‐based quality control strategy integrates sigma values (analytical performance) and clinical risk (potential severity of harm). The introduction of this approach enhances traditional quality control models by considering not only measurement imprecision but also the clinical consequences of laboratory testing errors. This dual approach effectively improves the comprehensiveness of quality control decision‐making [11].

Traditional quality control strategies primarily focus on analytical performance, particularly the assessment of sigma values. However, as noted by Çubukçu et al. [12], the clinical impact of errors plays a crucial role in quality control decisions. For instance, in high‐risk tests, even if sigma values fall within acceptable ranges, the potential for severe consequences necessitates increased quality control frequency. By incorporating clinical risk, our strategy ensures that quality control decisions are more comprehensive and patient‐centered.

Additionally, although lyophilized quality control materials were used and transported under 2°C–8°C cold‐chain conditions by the NCCL, subtle differences in sample handling—such as incomplete reconstitution, temperature fluctuations during storage, or inconsistent homogenization—might have introduced minor variations affecting analytical imprecision and bias. These variations, while minimized through standardization, still underscore the importance of pre‐analytical consistency. Future multicenter evaluations may benefit from further harmonization of sample handling protocols and on‐site supervision to reduce potential biases and improve the comparability of sigma values across laboratories.

Our study revealed that 47.2% of the QGI values for special protein assays were below 0.8, indicating substantial deficiencies in analytical imprecision. This outcome is particularly concerning as imprecision is a fundamental requirement for obtaining reliable and reproducible results. Similar findings have been reported by other authors who found that many clinical laboratory assays failed to meet required imprecision thresholds, leading to erroneous results and potential misdiagnosis [24, 25]. In our case, this suggests that many laboratories may need to prioritize improvements in internal quality control systems and invest in better staff training and equipment calibration to meet the recommended imprecision benchmarks. The QGI below 0.8 suggests that the current imprecision is inadequate for clinical use, and further studies are needed to investigate the root causes of this variation. These may include equipment malfunctions, operator errors, or insufficient quality control practices.

In contrast to imprecision, only 25.0% of the projects achieved a QGI greater than 1.2, indicating that trueness in many of the analyzed assays was suboptimal. Previous studies have shown that trueness is often a limiting factor in clinical laboratory performance [26, 27]. Deficient trueness in laboratory testing can result in misdiagnosis, inappropriate treatment plans, and ultimately harm to patients. This study found that analytes such as ASO required improvements in both imprecision and trueness, while RF primarily needed trueness improvements. These findings corroborate the work of Luo et al. [28], who identified the need for tailored quality control interventions based on the specific performance deficiencies of each analyte. Laboratories should focus on enhancing both imprecision and trueness for analytes where deficiencies in either parameter are identified, as improving one aspect without the other can lead to incomplete improvements in overall performance.

A key contribution of this study is the development of individualized risk‐based SQC strategies, based on the Westgard sigma rules. This customization of quality control practices according to the specific sigma value of each analyte offers a more nuanced approach than traditional one‐size‐fits‐all methods. For instance, analytes with a sigma value less than 6 were identified as requiring tailored improvements. This is particularly useful in real‐world clinical settings, where resources may be limited, and targeted interventions are needed to optimize laboratory performance. Similar risk‐based SQC strategies have been advocated by Westgard [29], who emphasized that custom solutions based on sigma metrics are more efficient and cost‐effective than generalized quality control procedures. Our results suggest that the adoption of this approach can lead to significant improvements in laboratory performance, as demonstrated by the improved sigma metrics following the implementation of the tailored quality control strategies.

The implementation of a Sigma‐based QC strategy led to significant improvements in laboratory performance. By applying a customized SQC program, both the analytical performance and accuracy of key tests were enhanced, with Sigma values for 15% of the tests increasing by ≥ 2 units. The risk‐based strategy made quality control interventions more targeted.

In high‐throughput laboratories, the feasibility of running different levels of QC and processing large numbers of samples is an important consideration. While high‐throughput environments handle a substantial volume of samples, ensuring that each sample meets stringent quality control standards remains crucial. Different levels of quality control are applied based on the complexity of the tests and their clinical significance, with high‐risk tests requiring more frequent and stringent QC checks.

In practice, implementing multiple levels of quality control in high‐throughput laboratories requires overcoming challenges such as time, resources, and sample volume. Solutions such as automation, batch testing, and the use of control materials can make this process more feasible. Automation enables laboratories to scale up without compromising quality, while batch testing and control materials help reduce the burden of testing each sample individually.

To systematically enhance laboratory performance, it is essential to focus on targeted improvements within the “5M + E” framework—comprising man, machine, material, method, and environment. Human resources require continuous training and performance evaluations to elevate skill levels and ensure accountability. Equipment needs consistent maintenance and regular updates to maintain operational efficiency. Materials used, including reagents, must be of high quality and handled according to strict protocols to maintain their efficacy. Methods should be standardized and validated rigorously to reduce variability and improve the reliability of test results. Finally, the environment should be meticulously controlled to optimize conditions such as temperature and humidity, while ensuring the lab layout promotes safety and operational efficiency. By strategically addressing each of these components, laboratories can achieve marked improvements in the imprecision and accuracy of their assays, thereby enhancing the reliability and reproducibility of their results.

This study provides valuable insights into the application of six sigma methodology in laboratory quality management; however, several limitations should be acknowledged. First, the analysis was primarily based on IQC data, which may not adequately reflect external variables such as sample heterogeneity, reagent batch variation, and environmental influences, as noted by Li et al. [30]. Future research should consider incorporating EQA data or inter‐laboratory comparison results to enhance generalizability. Second, bias was not evaluated using certified reference materials or reference measurement procedures. Instead, it was approximated using EQA samples, which serve only as a surrogate for true bias estimation. Although five replicate measurements were conducted to minimize random error, the target value of the analyte was derived from peer group means rather than true reference values, which may affect the accuracy of bias estimation. Third, while the study proposed risk‐based quality control strategies, their practical implementation and long‐term effectiveness were not systematically assessed. Further research is warranted to validate these approaches in routine laboratory settings [31]. Lastly, the study did not address the potential impact of human factors, such as staff training, competency, and adherence to standard operating procedures, which may significantly influence laboratory performance. Future investigations should explore these variables to provide a more comprehensive understanding of quality determinants in clinical laboratories.

## 5 Conclusions

In conclusion, the six sigma approach provides a structured, data‐driven framework for improving analytical quality in clinical laboratories. This study highlights the utility of sigma metrics in identifying performance gaps in precision and accuracy, thereby supporting the development of targeted, risk‐based quality control strategies. Despite the promising findings, addressing current methodological limitations is essential to optimize implementation. Future research should focus on integrating six sigma with broader quality management systems to promote continuous improvement in laboratory performance and patient outcomes.

## Author Contributions

**Ming Hu:** investigation, methodology, writing and editing. **Huan Yang:** methodology, resources. **Jiaping Wang:** data curation. **Tingting Gao:** investigation, resources. **Sugang Zang:** investigation. **Jian Zeng:** investigation. **Fumeng Yang:** funding acquisition, project administration, supervision, investigation, formal analysis, data curation, review and editing.

## Conflicts of Interest

The authors declare no conflicts of interest.

## Data Availability

The data that support the findings of this study are available from the corresponding author upon reasonable request.
